# Central Adiposity and Tear Film Instability in Adults With Obesity

**DOI:** 10.1155/joph/7742500

**Published:** 2026-11-06

**Authors:** Ao Li, Yifei Wu, Rongteng Lin, Yubo Wu, Qian Nie, Lei Tian, Ying Jie

**Affiliations:** ^1^ Beijing Institute of Ophthalmology Beijing Tongren Eye Center Beijing Tongren Hospital Beijing Ophthalmology & Visual Sciences Key Laboratory Capital Medical University Beijing 100730 China ccmu.edu.cn; ^2^ School of Basic Medical Sciences Capital Medical University Beijing 100069 China ccmu.edu.cn; ^3^ Physical Examination Center Hebei General Hospital Shijiazhuang 050051 China hebmu.edu.cn

**Keywords:** noninvasive tear breakup time, obesity, ocular surface, tear film instability, waist-to-height ratio

## Abstract

**Purpose:**

To examine the association between central adiposity, assessed by waist‐to‐height ratio (WHtR), and tear film stability in adults with obesity.

**Methods:**

This cross‐sectional observational study included 269 adults with BMI ≥ 28 kg/m^2^. WHtR and noninvasive tear breakup time (NIBUT) were the primary exposure and outcome. Models adjusted for age, sex, education, systemic diseases, myopia, display exposure, smoking, alcohol consumption, and sleep problems. Secondary outcomes were Ocular Surface Disease Index (OSDI) and dry eye disease (DED) status; other adiposity indices were examined in separate models.

**Results:**

Each 1‐SD higher WHtR was associated with shorter NIBUT (adjusted *β* −0.341 s; 95% CI −0.612–−0.071; *p* = 0.014). The highest versus lowest WHtR tertile differed by −0.961 s (95% CI −1.696–−0.227). BMI had a numerically larger coefficient (−0.591 s; 95% CI −0.842–−0.339). DED was present in 208 participants (77.3%). The WHtR–DED association was nominally significant (OR 1.630; 95% CI 1.072–2.477; *p* = 0.022) but did not survive false discovery rate correction (*q* = 0.061).

**Conclusions:**

Higher WHtR was associated with modestly shorter NIBUT among adults with obesity, while BMI showed a numerically larger association. These findings support considering overall and abdominal adiposity in the clinical assessment of ocular surface function.

## 1. Introduction

Obesity has been linked to several ocular conditions, but its relationship with ocular surface function remains incompletely understood [1]. Dry eye disease (DED) affects ocular comfort and visual quality, with tear film instability central to the loss of ocular surface homeostasis [2, 3]. Adults with the same obesity classification may nevertheless differ in their symptoms and objective tear findings. Understanding the adiposity characteristics associated with these differences could help clinicians recognize systemic factors relevant to ocular surface assessment.

Studies of BMI and percentage body fat have reported mixed associations with dry eye symptoms and tear measurements [4–6]. Less is known about variation in objective tear film stability among adults who already meet an obesity criterion. This question matters clinically because an obesity diagnosis identifies a shared systemic condition but does not describe differences in its severity or abdominal distribution.

Waist‐to‐height ratio (WHtR) describes the abdominal size relative to stature and is readily obtained in clinical practice. It complements the weight‐based description of body size provided by BMI. Human studies linking visceral adipose tissue to inflammatory adipokine secretion provide a biological rationale for investigating abdominal adiposity [7]. High‐fat‐diet models have also demonstrated meibomian gland inflammation and changes in meibum composition, connecting metabolic disturbance with processes relevant to tear film stability [8, 9]. WHtR may, therefore, help characterize the relationship between body habitus and ocular surface function within obesity.

We studied 269 Chinese adults with obesity, using WHtR as the primary exposure and noninvasive tear breakup time (NIBUT) as the primary outcome. Continuous NIBUT captured variation in tear film stability, while Ocular Surface Disease Index (OSDI) score and DED status described symptoms and the combined symptom‐and‐sign outcome. We also examined BMI, waist circumference, and waist‐to‐hip ratio (WHR), and explored tear meniscus height (TMH), gland structure, and lipid status.

## 2. Materials and Methods

### 2.1. Participants and Eligibility Criteria

Adults with obesity attending routine employer‐sponsored or self‐funded health examinations at Hebei General Hospital, a tertiary public hospital in Shijiazhuang, China, between December 2024 and December 2025 were considered for inclusion. Participants voluntarily underwent additional ocular surface assessment without charge. Eligible participants were aged 18 years or older with BMI ≥ 28 kg/m^2^ and complete anthropometric measurements, questionnaire responses, lipid results, and ocular surface measurements from the same examination visit. Obesity was defined using the Chinese adult BMI criterion [10].

Exclusion criteria were inability to complete the required examinations; psychiatric or cognitive conditions preventing reliable participation; active ocular inflammation or infection; recent ocular or systemic surgery that could interfere with assessment; and systemic, physical, or mobility limitations preventing completion of the examination. Common chronic diseases, including diabetes, hypertension, thyroid disease, rheumatic disease, and allergic disease, were included as covariates. Each participant contributed one record. A total of 269 participants met the eligibility criteria and were included in the analyses (Supporting Figure S1).

The study was approved by the Medical Ethics Committee of Hebei General Hospital on October 24, 2024 ((2024) Scientific Research Ethics Review No. 384). Electronic informed consent was obtained for the examinations and research use of deidentified records. The study followed the Declaration of Helsinki and is reported according to STROBE [11].

### 2.2. Anthropometric Measures and Covariates

BMI was calculated as weight in kilograms divided by height in meters squared. WHtR was waist circumference divided by height, and WHR was waist circumference divided by hip circumference. Each index was standardized using its sample SD and entered into a separate model. Pairwise Pearson correlations and variance inflation factors (VIFs) were calculated to assess correlations among the adiposity indices and collinearity within the regression models.

Structured questionnaires recorded age, sex, education, self‐reported myopia category, daily video display terminal (VDT) exposure, smoking, alcohol consumption, sleep problems, and physician‐diagnosed diabetes, hypertension, thyroid disease, rheumatic disease, and allergic disease. Systemic disease history and sleep‐problem status were coded as binary variables. Myopia was categorized as absent, mild, moderate, or high. Multilevel covariates were represented by indicator variables; definitions, reference categories, and frequencies are given in Supporting Table S1.

Covariates were selected for their plausible relationships with adiposity or ocular surface status. Triglycerides (TG), total cholesterol (TC), HDL cholesterol (HDL‐C), and LDL cholesterol (LDL‐C) were obtained from routine blood tests. Hyperlipidemia history was omitted because it overlaps with measured lipid concentrations and the lipid‐based group definition. For the exploratory four‐group analysis, lipid abnormality was defined as TG ≥ 1.7 mmol/L or HDL‐C < 1.03 mmol/L in men or < 1.29 mmol/L in women [12].

### 2.3. Ocular Measurements and Outcome Definitions

Ocular surface assessment was performed after 10 min of room acclimation, in the order OSDI, TMH, NIBUT, and infrared meibography. Room temperature was 20.1 ± 0.5°C and relative humidity was 63.5 ± 6.2%, with consistent lighting and no direct airflow. Right‐eye measurements were used for all analyses to avoid intereye dependence.

TMH and NIBUT were measured with the Dry Eye Detector BVD6600 (Big Vision Medical Technology, Suzhou, China). Three consecutive automated acquisitions were obtained. The mean of the device‐reported average‐NIBUT values was used, and central TMH measurements were averaged over the three acquisitions. A previous comparison with the Keratograph 5M in 38 participants at Beijing Tongren Hospital provides supporting information on average‐NIBUT agreement [13]. Infrared meibography of the everted upper and lower lids was graded by one experienced examiner masked to anthropometric questionnaire information and lipid results. The meibography‐derived ordinal gland score ranged from 0 to 3 and was analyzed as a measure of gland structure.

Participants completed a Chinese translation of the 12‐item OSDI, which was scored on the standard 0–100 scale [14–16]. Continuous NIBUT was the primary outcome; OSDI score and DED status were secondary outcomes. DED was defined for this study as OSDI ≥ 13 together with NIBUT < 10 s, combining symptoms with tear film instability within the TFOS DEWS symptom‐and‐sign framework [17]. The NIBUT measurement used for this definition was the device‐reported average value; diagnostic cutoffs can vary with the measurement technique [18].

### 2.4. Statistical Analysis

Multivariable linear regression examined NIBUT and OSDI, and logistic regression examined the DED status. Model 1 adjusted for age and sex. Model 2 additionally adjusted for education, diabetes, hypertension, thyroid disease, rheumatic disease, allergic disease, myopia, VDT exposure, smoking, alcohol consumption, and sleep‐problem status. Model 3 added TG and HDL‐C. A sensitivity model included all four measured lipids. Linear models used HC3 heteroscedasticity‐robust covariance and t‐based confidence intervals; logistic models used maximum‐likelihood estimation and Wald confidence intervals. All main models included 269 participants with complete values for the modeled variables; no imputation was required.

The primary WHtR–NIBUT association was assessed using its two‐sided *p* value. Benjamini–Hochberg correction was applied to the other 11 tests in the four‐index by three‐outcome Model 2 family, with *q* < 0.05 indicating significance after false‐discovery rate (FDR) correction [19]. Modified Poisson regression with robust covariance was used to estimate DED prevalence ratios in a sensitivity analysis [20].

WHtR tertiles were defined by cut points of 0.567,466 and 0.602,339, containing 90, 90, and 89 participants. Indicator models compared the second and third tertiles with the first; an ordinal score of 0, 1, or 2 assessed linear trend. Adjusted means and probabilities were estimated by marginal standardization over the observed covariates, with delta‐method confidence intervals. The exploratory four‐group analysis combined WHtR below or above its median (0.584,795) with lipid‐abnormality status.

Exploratory analyses examined TMH and gland score, including a fractional‐rank analysis of gland score. NIBUT sensitivity analyses used fractional ranks and a two‐part model, as described in the Supporting Methods. Sex‐related differences were examined in a pooled Model 2 model with a WHtR‐by‐sex interaction. Exploratory and sensitivity analyses used uncorrected *p* values. Analyses were performed in Python using NumPy, pandas, and SciPy.

## 3. Results

### 3.1. Participant Characteristics

The study included 269 adults with obesity (mean age 47.70 years, SD 12.75); 168 (62.5%) were men. Mean BMI was 30.68 kg/m^2^ (SD 2.68), and the WHtR SD was 0.04213. Mean NIBUT was 3.44 s (SD 2.33; median 2.1 s, IQR 2.0–4.2), mean OSDI was 25.23 (SD 12.75), and mean TMH was 0.296 mm (SD 0.109). The gland score median was 1 (IQR 0–2). DED was present in 208 participants (77.3%). Table 1 describes the participant characteristics across WHtR tertiles.

**TABLE 1:** Baseline characteristics across WHtR tertiles.

Characteristic	T1 (*n* = 90)	T2 (*n* = 90)	T3 (*n* = 89)
Age, years	43.70 ± 11.84	48.50 ± 12.71	50.92 ± 12.75
BMI, kg/m^2^	29.17 ± 1.36	30.04 ± 1.38	32.86 ± 3.24
Waist circumference, cm	94.60 ± 5.22	101.18 ± 3.92	108.93 ± 6.60
WHR	0.896 ± 0.043	0.943 ± 0.046	0.966 ± 0.055
WHtR	0.544 ± 0.018	0.586 ± 0.011	0.634 ± 0.029
NIBUT, s	4.05 ± 2.75	3.36 ± 2.19	2.92 ± 1.85
OSDI, points	25.16 ± 14.69	24.72 ± 11.63	25.82 ± 11.80
TMH, mm	0.313 ± 0.106	0.323 ± 0.111	0.250 ± 0.096
Gland score, median (IQR)	1 (0–2)	1 (0–2)	1 (0–1)
Men, *n* (%)	64 (71.1)	57 (63.3)	47 (52.8)
Diabetes, *n* (%)	5 (5.6)	7 (7.8)	13 (14.6)
Hypertension, *n* (%)	13 (14.4)	14 (15.6)	10 (11.2)
Sleep‐problem status, *n* (%)	39 (43.3)	44 (48.9)	39 (43.8)
VDT > 6 h/day, *n* (%)	41 (45.6)	37 (41.1)	36 (40.4)
TG, mmol/L	2.32 ± 2.08	2.25 ± 1.58	2.42 ± 3.14
HDL‐C, mmol/L	1.23 ± 0.22	1.21 ± 0.20	1.24 ± 0.23
DED, *n* (%)	63 (70.0)	70 (77.8)	75 (84.3)

*Note:* Values are mean ± SD unless specified. T1: WHtR ≤ 0.567,466; T2:  > 0.567,466 to ≤ 0.602,339; T3:  > 0.602,339. Tertiles were based on sample quantiles. NIBUT, noninvasive tear breakup time; WHtR, waist‐to‐height ratio; TG, triglycerides.

Abbreviations: BMI, body mass index; DED, dry eye disease; HDL‐C, HDL cholesterol; OSDI, Ocular Surface Disease Index; TMH, tear meniscus height; VDT, video display terminal; WHR, waist‐to‐hip ratio.

Anthropometric correlations ranged from 0.148 to 0.842, including WHtR–BMI *r* = 0.702 and WHtR–waist *r* = 0.842 (Supporting Table S2A). Exposure VIFs in Model 2 ranged from 1.074 to 1.224; the maximum covariate VIF was 2.846. The maximum VIF was 2.867 in Model 3 and 29.744 in the four‐lipid sensitivity models (Supporting Table S2B).

### 3.2. Main Associations and Model Progression

Each 1‐SD higher WHtR was associated with a 0.341 s reduction in NIBUT in Model 2 (95% CI −0.612–−0.071; *p* = 0.014). The coefficient remained negative from the unadjusted model through Models 1–3 (Table 2). WHtR was not clearly associated with OSDI (β 0.907 points; 95% CI −0.470–2.284; *p* = 0.196). Its DED association was nominal (OR 1.630; 95% CI 1.072–2.477; *p* = 0.022) but did not meet the secondary‐family FDR threshold (*q* = 0.061; Table 3 and Figure 1).

**TABLE 2:** WHtR association across model specifications.

Outcome	Unadjusted	Model 1	Model 2	Model 3
NIBUT β, s	−0.447 (−0.687, −0.207)	−0.383 (−0.633, −0.134)	−0.341 (−0.612, −0.071)	−0.338 (−0.611, −0.065)
	*p* < 0.001	*p* = 0.003	*p* = 0.014	*p* = 0.016

OSDI β, points	0.761 (−0.753, 2.276)	0.777 (−0.836, 2.389)	0.907 (−0.470, 2.284)	0.850 (−0.519, 2.218)
	*p* = 0.323	*p* = 0.344	*p* = 0.196	*p* = 0.223

DED OR	1.493 (1.092, 2.042)	1.431 (1.018, 2.011)	1.630 (1.072, 2.477)	1.692 (1.103, 2.595)
	*p* = 0.012	*p* = 0.039	*p* = 0.022	*p* = 0.016

*Note:* Effects per 1 SD higher WHtR (0.04213), with 95% CIs. Linear models use HC3 covariance and t inference; DED ORs use logistic Wald inference. Model 1: age and sex. Model 2: Model 1 plus education, diabetes, hypertension, thyroid disease, rheumatic disease, allergic disease, myopia, VDT exposure, smoking, alcohol consumption, and sleep‐problem status. Model 3: Model 2 plus TG and HDL‐C. All models include 269 participants. The four adiposity indices are entered in separate models.

Abbreviation: DED, dry eye disease.

**TABLE 3:** Main associations in separate Model 2 models.

Exposure	Outcome	Effect (95% CI)	*p*	FDR q
WHtR	NIBUT β, s	−0.341 (−0.612, −0.071)	0.014	Primary
WHtR	OSDI β, points	0.907 (−0.470, 2.284)	0.196	0.239
WHtR	DED OR	1.630 (1.072, 2.477)	0.022	0.061
BMI	NIBUT β, s	−0.591 (−0.842, −0.339)	< 0.001	< 0.001
BMI	OSDI β, points	1.592 (0.291, 2.893)	0.017	0.061
BMI	DED OR	2.444 (1.424, 4.194)	0.001	0.006
Waist circumference	NIBUT β, s	−0.200 (−0.417, 0.017)	0.071	0.130
Waist circumference	OSDI β, points	0.919 (−0.422, 2.261)	0.178	0.239
Waist circumference	DED OR	1.546 (1.034, 2.310)	0.034	0.074
WHR	NIBUT β, s	0.022 (−0.207, 0.251)	0.849	0.849
WHR	OSDI β, points	0.727 (−0.630, 2.085)	0.292	0.322
WHR	DED OR	1.298 (0.894, 1.885)	0.171	0.239

*Note:* Effects per sample SD in separate Model 2 models (*n* = 269). Model 2 adjusts for age, sex, education, diabetes, hypertension, thyroid disease, rheumatic disease, allergic disease, myopia, VDT exposure, smoking, alcohol consumption, and sleep‐problem status. The primary WHtR–NIBUT test was assessed with its *p* value; the remaining 11 tests constitute the secondary FDR family.

Abbreviations: DED, dry eye disease; FDR, false‐discovery rate.

**FIGURE 1:**
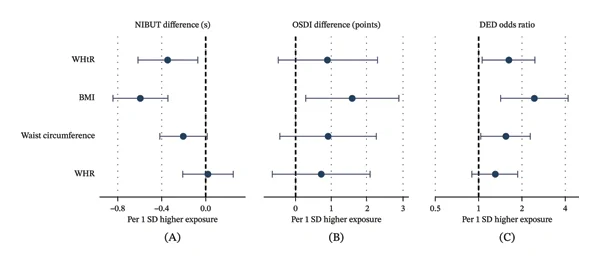
Associations of adiposity indices with ocular surface outcomes. Effects per 1 SD higher index in separate Model 2 models (*n* = 269), with 95% CIs. (A and B) show linear differences; (C) shows DED odds ratios on a log scale. The primary WHtR–NIBUT test has *p* = 0.014. Table 3 gives secondary *p* and *q* values. DED, dry eye disease. (A) NIBUT difference (s), (B) OSDI difference (points), and (C) DED odds ratio.

BMI had a numerically larger NIBUT coefficient in its separate Model 2 (−0.591 s per SD; 95% CI −0.842–−0.339; *p* < 0.001; *q* < 0.001) and was associated with DED after FDR correction (OR 2.444; 95% CI 1.424–4.194; *q* = 0.006). The BMI–OSDI association was nominal (*p* = 0.017; *q* = 0.061). Waist circumference had an NIBUT estimate of −0.200 s (95% CI −0.417–0.017; *p* = 0.071); its DED association was nominal (*p* = 0.034; *q* = 0.074). WHR showed no clear association with the three main outcomes.

### 3.3. WHtR Tertiles and Exploratory Groups

Adjusted NIBUT means were 3.945, 3.398, and 2.984 s across WHtR T1, T2, and T3. T3 versus T1 differed by −0.961 s (95% CI −1.696 to −0.227; *p* = 0.011); the ordinal‐trend *p* value was 0.010 (Table 4 and Figure 2). DED counts were 63/90, 70/90, and 75/89. The T3‐versus‐T1 DED OR was 2.774 (95% CI 1.064–7.232; *p* = 0.037), and the trend OR per tertile was 1.643 (95% CI 1.025–2.632; *p* = 0.039; Table 5). These secondary comparisons used uncorrected *p* values.

**TABLE 4:** Adjusted NIBUT across WHtR tertiles.

Tertile	*n*	Adjusted mean (95% CI), s	Difference vs. T1 (95% CI), s	*p*
T1	90	3.945 (3.412, 4.479)	Reference	—
T2	90	3.398 (2.954, 3.842)	−0.547 (−1.246, 0.152)	0.124
T3	89	2.984 (2.543, 3.425)	−0.961 (−1.696, −0.227)	0.011
Per‐tertile trend	269	—	−0.481 (−0.848, −0.114)	0.010

**FIGURE 2:**
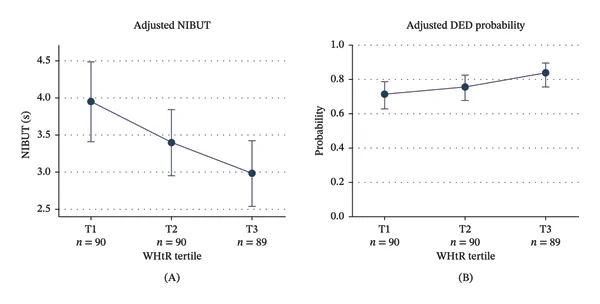
WHtR tertiles and adjusted ocular outcomes. Model 2 adjusted means (A) and marginal DED probabilities (B), with 95% CIs. Tertile cut points are 0.567,466 and 0.602,339. These secondary comparisons use uncorrected *p* values. DED, dry eye disease.

**TABLE 5:** Adjusted DED across WHtR tertiles.

Tertile	DED/*n*	Adjusted probability (95% CI)	OR vs. T1 (95% CI)	*p*
T1	63/90	0.718 (0.628, 0.793)	Reference	—
T2	70/90	0.761 (0.676, 0.829)	1.379 (0.594, 3.199)	0.454
T3	75/89	0.840 (0.759, 0.898)	2.774 (1.064, 7.232)	0.037
Per‐tertile trend	208/269	—	1.643 (1.025, 2.632)	0.039

*Note:* Both panels use Model 2 covariates, marginal standardization, and Table 1 cut points. Ordinal trend uses scores 0, 1, and 2. *p* values are uncorrected secondary results.

Abbreviation: DED, dry eye disease.

In the exploratory four‐group model, high WHtR without the specified lipid abnormality had a DED OR of 4.154 (95% CI 1.183–14.584; *p* = 0.026), and high WHtR with lipid abnormality had an OR of 3.725 (95% CI 1.282–10.824; *p* = 0.016), each relative to low WHtR without lipid abnormality (Table 6 and Figure 3).

**TABLE 6:** Exploratory WHtR and lipid groups.

Group	DED/*n*	OR (95% CI)	*p*	Adjusted probability (95% CI)
Low WHtR/no lipid abnormality	29/46	Reference	—	0.663 (0.537, 0.770)
Low WHtR/lipid abnormality	65/87	1.662 (0.606, 4.558)	0.323	0.736 (0.642, 0.812)
High WHtR/no lipid abnormality	40/48	4.154 (1.183, 14.584)	0.026	0.843 (0.728, 0.915)
High WHtR/lipid abnormality	74/88	3.725 (1.282, 10.824)	0.016	0.832 (0.749, 0.891)

*Note:* Low WHtR < 0.584,795; high WHtR ≥ 0.584,795. Lipid abnormality: TG ≥ 1.7 mmol/L or HDL‐C < 1.03 mmol/L in men or < 1.29 mmol/L in women. Model 2 covariates; *n* = 269. This exploratory analysis uses uncorrected *p* values.

Abbreviation: DED, dry eye disease.

**FIGURE 3:**
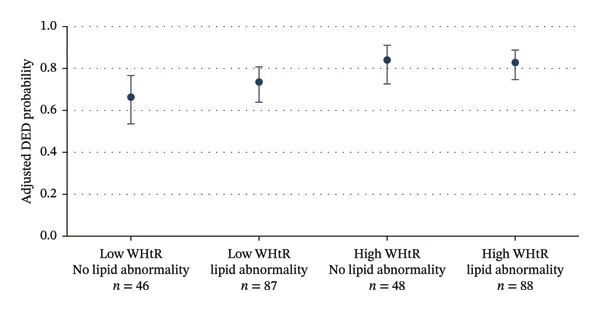
Adjusted DED probabilities across WHtR and lipid groups. Marginal probabilities and 95% CIs from the exploratory Model 2 analysis. The WHtR median split and lipid criteria are given in Table 5. DED, dry eye disease.

### 3.4. Sensitivity Analyses and Additional Outcomes

The WHtR DED prevalence ratio was 1.087 (95% CI 1.019–1.161; *p* = 0.012), and WHtR was associated with a lower NIBUT fractional rank (*p* = 0.018). The WHtR‐by‐sex interaction was imprecise (*p* = 0.096); the study was not adequately powered to establish a sex‐specific association (Supporting Table S3A). The four‐lipid NIBUT coefficient was −0.347 s (95% CI −0.625–−0.068; *p* = 0.015; Supporting Table S3B).

Higher WHtR was associated with lower TMH in the exploratory Model 2 (β −0.034 mm per SD; 95% CI −0.048–−0.020; *p* < 0.001). The ordinal gland‐score estimate was imprecise (−0.074 grade units; 95% CI −0.203–0.055; *p* = 0.260), and the fractional‐rank analysis likewise showed no clear association (Supporting Table S4).

## 4. Discussion

Higher WHtR was associated with shorter NIBUT among adults with obesity, with a consistent direction across adjustment sets and progressively lower adjusted NIBUT across WHtR tertiles. BMI also showed an association, with a numerically larger coefficient. Higher WHtR was associated with lower TMH in the exploratory analysis, whereas OSDI and structural gland grading showed no clear association. The findings indicate that differences in body habitus within obesity accompany differences in objective tear function.

A common obesity classification does not imply uniform ocular surface function. Previous studies of body fat and BMI have reported different relationships with dry eye symptoms and signs [4–6]. Our findings show that variation in adiposity remains relevant to tear film stability even after restricting the population to adults with obesity. BMI retained an association within this range, with an NIBUT coefficient of −0.591 s per SD, compared with −0.341 s for WHtR. Waist‐based measurements add a practical description of abdominal body habitus. Because the indices were correlated and modeled separately, the coefficient difference does not establish superiority or an additional effect of WHtR beyond BMI.

The absolute NIBUT differences were modest: 0.341 s per WHtR SD and 0.961 s between the highest and lowest tertiles. They occurred in a population with generally short breakup times, with adjusted means decreasing from 3.945 to 2.984 s across tertiles. These estimates help place differences in tear film stability in their systemic context. A clinically important change for an individual patient or a treatment threshold was not assessed.

WHtR was not clearly associated with OSDI, while its association with DED was nominally positive (OR 1.630; *p* = 0.022) and did not survive secondary‐family FDR correction (*q* = 0.061). The highest versus lowest WHtR tertile had higher DED odds (OR 2.774; 95% CI 1.064–7.232) in the uncorrected secondary comparison. The evidence was, therefore, strongest for continuous NIBUT. A binary cutoff compresses variation in tear film stability, while OSDI captures the patient’s experience of ocular surface disease. The different relationships with WHtR are consistent with the recognized variability in agreement between dry eye signs and symptoms [21]. Clinically, symptoms and objective tear measurements should be considered together when assessing an adult with obesity.

Adipose inflammation and altered lipid handling offer plausible links between obesity and tear film homeostasis. Visceral adipose tissue can release interleukin‐6 into the circulation [7], and elevated interleukin‐6 and tumor necrosis factor‐α have been reported in tears from patients with dry eye [22]. Experimental studies have shown high‐fat‐diet‐associated meibomian inflammation and changes in meibum lipid composition [8, 9, 23]. Such changes could alter lipid secretion and tear film stability without a corresponding difference in an imaging‐based gland grade. Tear film instability may in turn expose the epithelium to transient hyperosmolar stress and contribute to local inflammation and sensory stimulation [24, 25]. These pathways were not measured directly in this study.

The lower TMH associated with higher WHtR suggests that the aqueous tear reservoir also deserves attention. The Osaka study reported reduced tear volume in adults with metabolic syndrome although its exposure and tear‐volume assessment differed from ours [26]. A smaller tear reservoir could provide less aqueous support for a stable tear film; TMH itself does not directly measure lacrimal secretion. Reduced corneal nerve measures have also been described in severe obesity [27]. Changes in corneal sensation or reflex tearing could contribute to differences between objective findings and symptom reporting. Together with the gland‐score results, these observations support examining tear function alongside gland appearance.

Relative to low WHtR without the specified lipid abnormality, the DED OR was 4.154 (*p* = 0.026) for high WHtR without lipid abnormality and 3.725 (*p* = 0.016) for high WHtR with lipid abnormality. Both high‐WHtR groups had higher estimated DED odds, but the wide, overlapping confidence intervals do not establish a difference between them. The exploratory group findings and persistence of the NIBUT association after TG and HDL‐C adjustment suggest that these fasting lipid measures do not fully capture the tear‐function differences accompanying adiposity. Reported associations between dyslipidemia and dry eye are also heterogeneous [28]. Absence of the specified lipid abnormality therefore does not exclude adverse tear findings in an adult with obesity.

BMI and waist measurements can be recorded as part of the systemic history when assessing tear film instability in adults with obesity. Their associations with objective tear findings suggest that body habitus deserves attention alongside patient‐reported symptoms and gland appearance. Prospective follow‐up should determine whether changes in overall or abdominal adiposity track changes in tear function and whether anthropometric information adds clinical value beyond routine ocular surface assessment.

The continuous primary outcome and complementary tear assessments allowed examination of ocular surface function within a population sharing an obesity diagnosis. The cross‐sectional design limits inference about temporal direction and causality. The single‐center health‐examination setting and selection of complete records limit generalizability. Medication use, hormonal factors, disease control, and prior ocular treatment may contribute to residual confounding. Binary estimates were less precise because only 61 participants did not meet the DED definition relative to the 20 Model 2 slope parameters; the sex interaction was also underpowered.

## 5. Conclusions

Higher WHtR was associated with shorter NIBUT among adults with obesity, while BMI showed a numerically larger association. The exploratory TMH finding also suggests variation in the aqueous tear reservoir with body habitus. Overall and abdominal adiposity are relevant components of the clinical context of ocular surface assessment; longitudinal studies are needed to establish their value for individual patient monitoring.

## Author Contributions

Ao Li and Yifei Wu had full access to the analysis data and take responsibility for the analysis. Concept and design: Ao Li, Yifei Wu, Lei Tian, and Ying Jie. Acquisition, analysis, or interpretation: Ao Li, Yifei Wu, Rongteng Lin, Yubo Wu, and Qian Nie. Drafting: Ao Li, Yifei Wu, Rongteng Lin, and Yubo Wu. Critical revision: all authors. Statistical analysis: Ao Li and Yifei Wu. Administrative or technical support: Qian Nie, Lei Tian, and Ying Jie. Supervision: Lei Tian and Ying Jie.

## Funding

This study was supported by Beijing Hospitals Authority Ascent Plan (DFL20240202); Yicheng Outstanding Talents in Beijing Economic‐Technological Development Area.

## Ethics Statement

The study was approved by the Medical Ethics Committee of Hebei General Hospital on October 24, 2024 ((2024) Scientific Research Ethics Review No. 384). Electronic informed consent was obtained for the examinations and research use of deidentified records.

## Conflicts of Interest

The authors declare no conflicts of interest.

## Supporting Information

Additional supporting information can be found online in the Supporting Information section.

## Supporting information


**Supporting Information** Supporting 1. Supporting Methods. NIBUT processing and sensitivity analyses. Supporting 2. Figure S1. Study eligibility and analytic inclusion. Supporting 3. Table S1. Covariate definitions, coding, and frequencies. Supporting 4. Table S2A. Pearson correlations among anthropometric indices. Supporting 5. Table S2B. Variance inflation factors in the regression models. Supporting 6. Table S3A. Sensitivity analyses and sex interaction results. Supporting 7. Table S3B. WHtR associations after additional adjustment for four lipid measures. Supporting 8. Table S4. Exploratory associations of WHtR with tear meniscus height and meibography‐derived gland score.

## Data Availability

The deidentified data are available from the corresponding authors upon reasonable request.

## References

[1] Lam S. , Lindsey J. , Carranza Leon B. G. , and Takkouche S. , Shedding Light on Eye Disease in Obesity: A Review, Clinical Obesity. (2024) 14, no. 1, 10.1111/cob.12616. 37532290

[2] Craig J. P. , Nichols K. K. , Akpek E. K. et al., TFOS DEWS II Definition and Classification Report, Ocular Surface. (2017) 15, no. 3, 276–283, 10.1016/j.jtos.2017.05.008. 28736335

[3] Wolffsohn J. S. , Benítez-Del-Castillo J. M. , Loya-Garcia D. et al., TFOS DEWS III: Diagnostic Methodology, American Journal of Ophthalmology. (2025) 279, 387–450, 10.1016/j.ajo.2025.05.033. 40451408

[4] Ho K. C. , Jalbert I. , Watt K. , and Golebiowski B. , A Possible Association Between Dry Eye Symptoms and Body Fat: A Prospective, Cross-Sectional Preliminary Study, Eye Contact Lens. (2017) 43, no. 4, 245–252, 10.1097/ICL.0000000000000275. 27243357

[5] Surmacz H. U. , Cotlinski A. L. , Gehlen M. L. , Nisihara R. , and Skare T. L. , Dry Eye and Percentage of Body Fat: A Cross-Sectional Prospective Study, International Ophthalmology. (2021) 41, no. 5, 1855–1861, 10.1007/s10792-021-01747-8. 33629234

[6] Alanazi S. A. , Assessment of Tear Film in Subjects With a High Body Mass Index, Clinical Optometry. (2019) 11, 77–84, 10.2147/OPTO.S218109. PMC6666370 31440111

[7] Fontana L. , Eagon J. C. , Trujillo M. E. , Scherer P. E. , and Klein S. , Visceral Fat Adipokine Secretion is Associated With Systemic Inflammation in Obese Humans, Diabetes. (2007) 56, no. 4, 1010–1013, 10.2337/db06-1656. 17287468

[8] Bu J. , Zhang M. , Wu Y. et al., High-Fat Diet Induces Inflammation of Meibomian Gland, Investigative Ophthalmology & Visual Science. (2021) 62, no. 10, 10.1167/iovs.62.10.13. PMC8374999 34398199

[9] Osae E. A. , Bullock T. , Chintapalati M. et al., Obese Mice With Dyslipidemia Exhibit Meibomian Gland Hypertrophy and Alterations in Meibum Composition and Aqueous Tear Production, International Journal of Molecular Sciences. (2020) 21, no. 22, 10.3390/ijms21228772. PMC7699756 33233559

[10] Zhou B. F. and Cooperative Meta-Analysis Group of the Working Group on Obesity in China , Predictive Values of Body Mass Index and Waist Circumference for Risk Factors of Certain Related Diseases in Chinese Adults: Study on Optimal Cut-Off Points of Body Mass Index and Waist Circumference in Chinese Adults, Biomedical and Environmental Sciences. (2002) 15, no. 1, 83–96. 12046553

[11] von Elm E. , Altman D. G. , Egger M. et al., The Strengthening the Reporting of Observational Studies in Epidemiology (STROBE) Statement: Guidelines for Reporting Observational Studies, Lancet. (2007) 370, no. 9596, 1453–1457, 10.1016/S0140-6736(07)61602-X. 18064739

[12] Alberti K. G. M. M. , Eckel R. H. , Grundy S. M. et al., Harmonizing the Metabolic Syndrome: A Joint Interim Statement of the International Diabetes Federation Task Force on Epidemiology and Prevention; National Heart, Lung, and Blood Institute; American Heart Association; World Heart Federation; International Atherosclerosis Society; and International Association for the Study of Obesity, Circulation. (2009) 120, no. 16, 1640–1645, 10.1161/CIRCULATIONAHA.109.192644. 19805654

[13] Li A. , Hao Y. , Shi R. et al., Associations of Complete Blood Count-Derived Inflammatory Markers With Tear Film Instability: A Cross-Sectional Study, Contact Lens and Anterior Eye. (2026) 49, no. 6, 10.1016/j.clae.2026.102840. 42731253

[14] Schiffman R. M. , Christianson M. D. , Jacobsen G. , Hirsch J. D. , and Reis B. L. , Reliability and Validity of the Ocular Surface Disease Index, Archives of Ophthalmology. (2000) 118, no. 5, 615–621, 10.1001/archopht.118.5.615. 10815152

[15] Lin C. , Yang M. M. , and Zheng Q. , Development and Evaluation of Chinese Version Questionnaires of Ocular Surface Disease Index, Recent Adv Ophthalmol. (2013) 33, no. 1, 38–40, 10.13389/j.cnki.rao.2013.01.011.

[16] Zhang X. M. , Yang L. T. , Zhang Q. et al., Reliability of Chinese Web-Based Ocular Surface Disease Index Questionnaire in Dry Eye Patients: A Randomized, Crossover Study, International Journal of Ophthalmology. (2021) 14, no. 6, 834–843, 10.18240/ijo.2021.06.07. PMC8165610 34150537

[17] Wolffsohn J. S. , Arita R. , Chalmers R. et al., TFOS DEWS II Diagnostic Methodology Report, Ocular Surface. (2017) 15, no. 3, 539–574, 10.1016/j.jtos.2017.05.001. 28736342

[18] Lim J. , Wang M. T. M. , and Craig J. P. , Evaluating the Diagnostic Ability of Two Automated Non-Invasive Tear Film Stability Measurement Techniques, Contact Lens and Anterior Eye. (2021) 44, no. 4, 10.1016/j.clae.2020.08.006. 32919934

[19] Benjamini Y. and Hochberg Y. , Controlling the False Discovery Rate: A Practical and Powerful Approach to Multiple Testing, Journal of the Royal Statistical Society B. (1995) 57, no. 1, 289–300, 10.1111/j.2517-6161.1995.tb02031.x.

[20] Zou G. , A Modified Poisson Regression Approach to Prospective Studies With Binary Data, American Journal of Epidemiology. (2004) 159, no. 7, 702–706, 10.1093/aje/kwh090. 15033648

[21] McMonnies C. W. , Why the Symptoms and Objective Signs of Dry Eye Disease May Not Correlate, Journal of Optometry. (2021) 14, no. 1, 3–10, 10.1016/j.optom.2020.10.002. PMC7752964 33243674

[22] Yoon K. C. , Jeong I. Y. , Park Y. G. , and Yang S. Y. , Interleukin-6 and Tumor Necrosis Factor-Alpha Levels in Tears of Patients With Dry Eye Syndrome, Cornea. (2007) 26, no. 4, 431–437, 10.1097/ICO.0b013e31803dcda2. 17457192

[23] Osae E. A. , Steven P. , Redfern R. et al., Dyslipidemia and Meibomian Gland Dysfunction: Utility of Lipidomics and Experimental Prospects With a Diet-Induced Obesity Mouse Model, International Journal of Molecular Sciences. (2019) 20, no. 14, 10.3390/ijms20143505. PMC6678820 31319467

[24] Liu H. , Begley C. , Chen M. et al., A Link Between Tear Instability and Hyperosmolarity in Dry Eye, Investigative Ophthalmology & Visual Science. (2009) 50, no. 8, 3671–3679, 10.1167/iovs.08-2689. 19324847

[25] Bron A. J. , de Paiva C. S. , Chauhan S. K. et al., TFOS DEWS II Pathophysiology Report, Ocular Surface. (2017) 15, no. 3, 438–510, 10.1016/j.jtos.2017.05.011. 28736340

[26] Kawashima M. , Uchino M. , Yokoi N. et al., Decreased Tear Volume in Patients With Metabolic Syndrome: The Osaka Study, British Journal of Ophthalmology. (2014) 98, no. 3, 418–420, 10.1136/bjophthalmol-2013-303953. PMC3932960 24344231

[27] Iqbal Z. , Kalteniece A. , Ferdousi M. et al., Corneal Keratocyte Density and Corneal Nerves Are Reduced in Patients With Severe Obesity and Improve After Bariatric Surgery, Investigative Ophthalmology & Visual Science. (2021) 62, no. 1, 10.1167/iovs.62.1.20. PMC7817877 33475689

[28] Wang T. H. , Tsai Y. J. , Wang Y. H. , Wu C. L. , and Lin I. C. , Relationship Between Dry Eye Disease and Dyslipidemia: A Systematic Review, Journal of Clinical Medicine. (2023) 12, no. 20, 10.3390/jcm12206631. PMC10607063 37892769

