# Supplementary material for: Central Adiposity and Tear Film Instability in Adults With Obesity
Source: J Ophthalmol. 2026 Nov 6;2026:7742500. doi: 10.1155/joph/7742500 (PMC13740966; doi:10.1155/joph/7742500)
Supplement: Supplementary file 1 [file JOPH-2026-7742500-s001.docx]

**Supplementary material**

Central Adiposity and Tear Film Instability in Adults with Obesity

# **Study eligibility and Supplementary Methods**

**Figure S1** Study eligibility and analytic inclusion. Records were eligible if age was ≥ 18 years, BMI was ≥ 28 kg/m², and the anthropometric, questionnaire, lipid, and ocular surface measurements were complete. Each of the 269 included participants contributed one record, and all main regression models used 269 participants.

## **NIBUT sensitivity analyses**

NIBUT values above 10 s were recorded as 10 s. Sensitivity analyses used fractional ranks and a two-part model comprising logistic regression for NIBUT > 2 s and linear regression among the 139 participants with NIBUT > 2 s (**Table S3A**).

# **Covariate definitions and coding**

**Table S1 Model covariates**

| **Variable** | **Operational coding** | **Counts in code order** |
| --- | --- | --- |
| Age | Continuous years | No missing values |
| Sex | 0 male (reference); 1 female | 168; 101 |
| Education | 0 junior high or below (reference); 1 senior high or vocational secondary; 2 college or above | 35; 110; 124 |
| Myopia | 0 none (reference); 1 mild; 2 moderate; 3 high, self-reported categories; refractive thresholds unavailable | 130; 63; 60; 16 |
| VDT | 0 < 3 h/day; 1 3–6 h/day (reference); 2 > 6 h/day | 8; 147; 114 |
| Smoking | 0 nonsmoker (reference); 1 1–7 cigarettes/day; 2 > 7 cigarettes/day | 153; 64; 52 |
| Alcohol | 0 nondrinker (reference); 1 < 3 times/week; 2 ≥ 3 times/week | 147; 74; 48 |
| Sleep-problem status | 0 absent (reference); 1 present. Self-reported binary status | 147; 122 |
| Diabetes | 0 absent (reference); 1 self-reported physician diagnosis | 244; 25 |
| Hypertension | 0 absent (reference); 1 self-reported physician diagnosis | 232; 37 |
| Thyroid disease | 0 absent (reference); 1 self-reported physician diagnosis | 231; 38 |
| Rheumatic disease | 0 absent (reference); 1 self-reported physician diagnosis; subtype unavailable | 261; 8 |
| Allergic disease | 0 absent (reference); 1 self-reported physician diagnosis; subtype unavailable | 261; 8 |

Multilevel variables were represented by indicator variables. VDT exposure of 3–6 h/day was the reference category because fewer than 3 h/day included only eight observations. Systemic disease histories were self-reported physician diagnoses. All modeled variables were complete in the 269 included records.

# **Correlations and collinearity**

**Table S2A Pearson correlations of anthropometric indices**

| **Index** | **WHtR** | **BMI** | **Waist** | **WHR** |
| --- | --- | --- | --- | --- |
| WHtR | 1.000 | 0.702 | 0.842 | 0.548 |
| BMI | 0.702 | 1.000 | 0.656 | 0.148 |
| Waist | 0.842 | 0.656 | 1.000 | 0.685 |
| WHR | 0.548 | 0.148 | 0.685 | 1.000 |

**Table S2B VIFs in separate exposure models**

| **Exposure** | **Exposure VIF in Model 2** | **Maximum VIF in Model 2** | **Maximum VIF in Model 3** | **Maximum VIF with all four lipids** |
| --- | --- | --- | --- | --- |
| WHtR | 1.201 | 2.827 | 2.847 | 29.477 |
| BMI | 1.224 | 2.846 | 2.867 | 29.695 |
| Waist | 1.104 | 2.773 | 2.796 | 29.744 |
| WHR | 1.074 | 2.747 | 2.766 | 29.476 |

Each model includes one adiposity index. Model 3 adds TG and HDL-C; the sensitivity model includes TG, TC, HDL-C, and LDL-C together. The maximum four-lipid VIF was 29.744, reflecting redundancy among the lipid measurements.

# **Sensitivity and sex interaction results**

**Table S3A Distribution and model sensitivities**

| **Exposure** | **Outcome or analysis** | **Effect (95% CI)** | **P** | **n** |
| --- | --- | --- | --- | --- |
| WHtR | DED prevalence ratio | PR 1.087 (1.019, 1.161) | 0.012 | 269 |
| BMI | DED prevalence ratio | PR 1.123 (1.048, 1.203) | 0.001 | 269 |
| Waist | DED prevalence ratio | PR 1.065 (1.003, 1.132) | 0.041 | 269 |
| WHR | DED prevalence ratio | PR 1.045 (0.987, 1.107) | 0.134 | 269 |
| WHtR | NIBUT > 2 s | OR 0.689 (0.509, 0.933) | 0.016 | 269 |
| WHtR | NIBUT in values > 2 s | β -0.292 (-0.836, 0.252) | 0.290 | 139 |
| WHtR | Fractional rank of NIBUT | β -0.039 (-0.072, -0.007) | 0.018 | 269 |
| WHtR by sex | NIBUT interaction | β 0.400 (-0.072, 0.871) | 0.096 | 269 |
| WHtR | NIBUT slope in men | β -0.503 (-0.884, -0.121) | 0.010 | 269 |
| WHtR | NIBUT slope in women | β -0.103 (-0.426, 0.220) | 0.530 | 269 |

All models use Model 2 covariates. Modified Poisson PRs use robust HC1 sandwich covariance. Linear and rank models use HC3 covariance and t inference; logistic ORs use Wald inference. Fractional ranks are average ranks divided by n. The conditional NIBUT analysis includes 139 participants. Sex slopes are contrasts from one pooled model (n = 269) with a WHtR-by-sex interaction and shared covariate coefficients. There are 168 men and 101 women. Exposure effects are per full-sample SD where applicable. These exploratory analyses use uncorrected P values.

**Table S3B Four-lipid sensitivity for WHtR**

| **Outcome** | **Effect (95% CI)** | **P** |
| --- | --- | --- |
| NIBUT β, s | -0.347 (-0.625, -0.068) | 0.015 |
| OSDI β, points | 0.834 (-0.553, 2.221) | 0.237 |
| DED OR | 1.745 (1.130, 2.695) | 0.012 |

Model 2 plus TG, TC, HDL-C, and LDL-C; n = 269. The maximum VIF was 29.744, reflecting redundancy among the lipid measurements. Exposure estimates are reported as sensitivity analyses.

# **Additional ocular outcomes**

**Table S4 Exploratory WHtR associations**

| **Outcome** | **β per 1 SD WHtR (95% CI)** | **P** |
| --- | --- | --- |
| TMH, mm | -0.034 (-0.048, -0.020) | < 0.001 |
| Ordinal gland score, grade units | -0.074 (-0.203, 0.055) | 0.260 |
| Gland score fractional rank | -0.017 (-0.055, 0.021) | 0.367 |

Model 2, n = 269; linear HC3 inference. The numeric gland-score model assumes equal spacing between grades; the fractional-rank analysis relaxes that assumption. Observed grades were 0 (n = 104), 1 (n = 91), 2 (n = 55), and 3 (n = 19). The score describes gland structure. P values are uncorrected.
